# Research Design and Statistical Methods in Indian Medical Journals: A Retrospective Survey

**DOI:** 10.1371/journal.pone.0121268

**Published:** 2015-04-09

**Authors:** Shabbeer Hassan, Rajashree Yellur, Pooventhan Subramani, Poornima Adiga, Manoj Gokhale, Manasa S. Iyer, Shreemathi S. Mayya

**Affiliations:** Department of Statistics, Level 6, Health Science Library Building, Manipal University, Manipal, 576104, Karnataka, India; Universiteit Gent, BELGIUM

## Abstract

Good quality medical research generally requires not only an expertise in the chosen medical field of interest but also a sound knowledge of statistical methodology. The number of medical research articles which have been published in Indian medical journals has increased quite substantially in the past decade. The aim of this study was to collate all evidence on study design quality and statistical analyses used in selected leading Indian medical journals. Ten (10) leading Indian medical journals were selected based on impact factors and all original research articles published in 2003 (N = 588) and 2013 (N = 774) were categorized and reviewed. A validated checklist on study design, statistical analyses, results presentation, and interpretation was used for review and evaluation of the articles. Main outcomes considered in the present study were – study design types and their frequencies, error/defects proportion in study design, statistical analyses, and implementation of CONSORT checklist in RCT (randomized clinical trials). From 2003 to 2013: The proportion of erroneous statistical analyses did not decrease (*χ^^2^^*=0.592, *Φ=*0.027, *p*=0.4418), 25% (80/320) in 2003 compared to 22.6% (111/490) in 2013. Compared with 2003, significant improvement was seen in 2013; the proportion of papers using statistical tests increased significantly (*χ^2^*=26.96, *Φ=*0.16, *p*<0.0001) from 42.5% (250/588) to 56.7 % (439/774). The overall proportion of errors in study design decreased significantly (*χ^2^*=16.783, *Φ=*0.12 *p*<0.0001), 41.3% (243/588) compared to 30.6% (237/774). In 2013, randomized clinical trials designs has remained very low (7.3%, 43/588) with majority showing some errors (41 papers, 95.3%). Majority of the published studies were retrospective in nature both in 2003 [79.1% (465/588)] and in 2013 [78.2% (605/774)]. Major decreases in error proportions were observed in both results presentation (*χ^2^*=24.477, *Φ=*0.17, *p*<0.0001), 82.2% (263/320) compared to 66.3% (325/490) and interpretation (*χ^2^*=25.616, *Φ=*0.173, *p*<0.0001), 32.5% (104/320) compared to 17.1% (84/490), though some serious ones were still present. Indian medical research seems to have made no major progress regarding using correct statistical analyses, but error/defects in study designs have decreased significantly. Randomized clinical trials are quite rarely published and have high proportion of methodological problems.

## Introduction

Good quality medical research generally requires not only an expertise in the chosen medical field of interest but also a sound knowledge of statistical methodology. Since, medical practitioners and policy makers often depend on the correct conclusions obtained from these research papers, conducting the correct statistical analysis is of paramount importance. Hence, statistical methodology has often been checked for predominantly in Western journals [1–12]. From 1970s, Altman et al. [13–17] have continued to study errors/defects in several aspects of study design and statistical methodologies used in medical journals. Afterwards, a series of publication guidelines have been proposed, including CONSORT and STROBE statements [18–24]. These guidelines have in general led to some improvement in the quality of publications in clinical research worldwide.

But such checking for statistical inconsistencies is quite rare and is indeed absent in Indian journals. Previously, Goyal et. al., [25] reported in an analysis of 46 clinical trials published in three journals—Indian Pediatrics, Indian Journal of Pharmacology, Journal of Postgraduate Medicine within the period of 2007–2008, that majority of papers had errors during the interpretation of results of these trials. This was found due to multiple treatment groups, repeated measurements of endpoints, multiple tests of significance, over reliability on P value and less use of confidence interval. Another paper by Jaykaran et. al., [26] analysed papers published in an 8 year span in two Indian journals—Indian Journal of Pharmacology and Indian Journal of Physiology and Pharmacology. Out of 196 papers which were analysed 150 papers had inappropriate descriptive methods, with the most common error being that Mean±SEM (standard error of mean) was used in the place of “mean (SD)” or “mean ± SD.” SEM generally quantifies uncertainty in estimate of the descriptive measure (like mean) whereas SD indicates dispersion of the data from the central tendency (like mean) [27]. As readers are generally interested in knowing the variability within sample, descriptive data should be precisely summarized with SD. About 600 biomedical and life science journals are published every year in India [28]. But if we take a look at the indexing statistics it presents a bleak picture. To take an example, out of the 5500 plus journals within PubMed database, only 39 (0.71%) are from India [28]. Similarly [28], in another database—EMBASE, number of Indian journals indexed in it is 128 (1.71%).

As more than 600 medical journals are being used within India by a large section of clinicians and readers, it has become increasingly more important to know and understand whether the statistical methodology used in Indian medical journals are suitable and to ascertain what all changes should be implemented for their improvement. In this article, we have reviewed original research articles published in 10 leading Indian medical journals (based on 2013 Impact Factors published by Thomson Reuters [29]) in 2003 and 2013. We determined the frequency of study designs, statistical methods and results presentation/ interpretation used and the most prevalent statistical errors in these research articles. We have also examined all the RCT’s (randomized clinical trials) published in these journals according to the CONSORT statement. In addition, we also discuss certain remedies which can lead to improvement of the publishing quality.

## Materials and Methods

### Journals

For this article we selected 10 leading medical journals published in India, and indexed by PubMed. The 10 journals are: Indian Journal of Medical Research, Indian Journal of Medical Sciences, Indian Journal of Otolaryngology & Head and Neck Surgery, The Indian Journal of Chest Diseases & Allied Sciences, Journal of Postgraduate Medicine, Indian Journal of Dermatology, Venereology, Leprology, Indian Journal of Cancer, Neurology India, Indian Pediatrics and Indian Journal of Ophthalmology (S1 Table).

All the original research articles published in these journals were peer reviewed. As majority of guidelines have come up during the past decade or so after the millennium, we intended to check how the Indian medical journals fare just before the introduction of many such reporting guidelines (for example, STROBE, STARD, SAMPL, TREND etc) and a decade after. This duration of a decade has seen the release of many such guidelines and hence many journals would adopt these guidelines leading to a significant change in the reporting errors. Hence, we examined all the original research articles published in 2003 and a decade later in 2013, as we would have access to all the articles in both these years. The present article included—study design types, frequencies of statistical methods, errors/defects in study design and statistical analyses, and also the implementation of CONSORT checklist in RCT’s (randomized clinical trials).

### Quality control

A strict and high quality control was maintained throughout the entire study. For the quality standards of the different study designs we used the checklists—CONSORT, STROBE, STARD, SAMPL, STREGA, PRISMA, SPIRIT and TREND [18–24]. In the early 1990’s Iain Chalmers said that, *“Failure to publish an adequate account of a well-designed clinical trial is a form of scientific misconduct that can lead those caring for patients to make inappropriate treatment decisions.”* [39].

The guidelines and checklists for reporting of scientific research are important documents which are meant to increases the transparency of reported research work and hence practically are meant to improve upon the conduct of research itself. Ever since the CONSORT (Consolidated Standards of Reporting Trials) statement was published in 1996, as a set of guidelines for basic reporting of clinical trials, these guidelines have significantly improved and several extensions have been proposed for various other forms of clinical trials. RCTs are considered to be in the top of evidence chain but it’s not always ethical to conduct them in every setting. Over these years many other guidelines or checklists have been coming up to reflect the other kinds of research studies like epidemiological (STROBE) [21], diagnostic (STARD) [20], meta-analysis (PRISMA) [23], statistical methodology (SAMPL) [24] etc.

The STROBE (Strengthening of Reporting of Observational Studies in Epidemiology) checklist consists of 22 items that should be included three kinds of analytic epidemiology studies: cross-sectional cohort and case-control studies. The aim of the checklist is to help researchers report the observational studies well.

CONSORT (Consolidated Standards of Reporting Trials) statement is a 25 item checklist which consists of recommendations for reporting all kinds of randomized trials. It gives the authors a standard method to prepare reports of randomised trials in a transparent manner which would then lead to a critical evaluation and interpretation.

STARD (Standards for the reporting of Diagnostic Accuracy Studies) consist of a checklist of 25 items which aims to improve the completeness of diagnostic accuracy studies which will allow the readers to assess any biases present in the study and to evaluate how it is generalizable when compared to other studies.

SAMPL (Statistical Analyses and Methods in the Published Literature) is a checklist used for assessing the completeness of basic statistical reporting for articles published in any biomedical journals.

The PRISMA (Preferred Reporting Items for Systematic Reviews and Meta-Analyses) statement is a 27 item checklist which can be used for evaluating the reporting in systematic reviews and meta-analyses. It aims to help the authors to report systematic reviews and meta-analyses in a complete manner.

STREGA (Strengthening the reporting of genetic association studies) is a checklist which aims to serve as a guiding tool for complete and unbiased reporting of gene-disease association studies which greatly helps in the evidence synthesis and bias investigation in the studies.

The TREND statement has a 22-item checklist which has been specifically developed to guide the reporting of all kinds of nonrandomized controlled trials. This statement complements the Consolidated Standards of Reporting Trials (CONSORT) statement which had been developed earlier for reporting of randomized controlled trials.

SPIRIT (Standard Protocol Items: Recommendations for Interventional Trials) is a checklist aiming to aid the improvement of all the clinical trial protocols.

Hence any study on understanding the progress of scientific journals would need to incorporate these above checklists as a means of truly gauging the transparency of the research work published and also the conduct of those studies.

All the statistical methods used in an article were counted but if one method is used multiply then it was counted only once. The preliminary checklist was derived from a combination of two other checklists [33–34] and after consulting various other publishing standards as mentioned above. The preliminary checklist was then subjected to discussion rounds and was later validated by three independent statistical researchers to come up with a well-defined checklist (S1 Appendix).

We also examined the RCT’s (randomized clinical trials) published in the journals via the CONSORT statement. Ten researchers participated in this study: 3 professors, 1 lecturer, and 6 postgraduates. All of them have had formal training in biostatistics and some of them have been in long-term teaching and research roles. Each article used in the study was reviewed by 3 participants independently, which included a professor with two postgraduates. Any discrepancies were noted and later solved by a group discussion.

### Data analysis

Since three researchers independently reviewed the articles, a senior professor randomly sampled 50 articles from each to assess for an agreement among them. A simple multi-rate Kappa test after Fleiss [38] was conducted and was found to be (k = 0.84) which indicated almost perfect agreement.

An a-priori power analyses conducted for 5% level of significance, 80% power for a medium effect size (*Φ* = 0.3) found a sample size of 82 for each year we selected for our study. For a small effect size (*Φ* = 0.1) at 5% level of significance with 80% power a sample size of 600 per year was obtained. Hence, we used no sampling procedure in our study but included all the original articles published by the top 10 Indian medical journals in 2003 and 2013. However, a post-hoc power analysis was not conducted since post-hoc power analyses is increasingly considered to be unnecessary. Such analyses are not required at all since if no significance is found, then there wasn’t sufficient power to detect the difference [35].

Epidata3.1 was used for double data entry and management. SPSS 16 and GraphPad were used for subsequent analysis, as appropriate. Chi-square and Fisher’s exact tests, (wherever appropriate) were used to compare any proportional differences in use of statistical methods and various errors/defects during the 10 years in the study period. All the tests used in this article were conducted at 5% level of significance and were 2-tailed. Chi-square tests were conducted at one degree of freedom.

## Results

### Study Design

As shown in S1–S2 Tables from 2003 to 2013, both the numbers of issues and articles in the 10 selected Indian medical journals were found to have increased (issues: 73 to 82; articles: 588 to 774). The basic science studies have remained same (*χ* ^*2*^ = 0.204, *Φ =* 0.042, *p* = 0.6518), 8.8% (52/588) in 2003 compared to 8.01% (62/774) in 2013. Though, the number of clinical trials have remained low (randomized clinical trials: 7.3%, (43/588) compared to 5.3% (41/774); nonrandomized clinical trials: 4.6% (27/588) compared to 6.98% (54/774)). The majority of the published original research articles have however remained retrospective, 79.1% (465/588) in 2003 and 78.2% (605/774) in 2013. However, the overall proportion of errors in study design has decreased significantly (*χ* ^*2*^ = 16.783, *Φ =* 0.12, *p*<0.0001), 41.3% (243/588) compared to 30.6% (237/774). In general, randomized clinical trials, nonrandomized clinical trials, cohort study and case-control study were seen to use statistical analyses more frequently in both years. A significant increase (*χ* ^*2*^ = 8.04, *Φ =* 0.32, *p* = 0.0046) was seen in randomized clinical trials being registered on the domestic or international clinical trial registries in 2013[58.5% (24/41)] as compared to 2003 [27.9% (12/43)]. As the current checklist used in the study is derived from CONSORT, the proportion of errors in randomized clinical trials have decreased significantly (*χ* ^*2*^ = 7.892, *Φ =* 0.31, *p* = 0.0065), 95.3% (41/43) in 2003 compared to 73.1% (30/41) in 2013, though only small number of randomized clinical trials we published as compared to retrospective studies. Omission of sample size estimation, failure to use (or report) randomization, failure to use (or report) blinding, and unclear primary outcome measures were the most common errors/defects in randomized clinical trials design (S3 Table). But the proportion with errors in sample size estimation did not improve (*χ* ^*2*^ = 3.44, *Φ =* 0.21, *p* = 0.0636), 90.7% (39/43) compared to 75.6% (31/41); As in S3 Table, more than one-half of the published articles still did not meet this important requirement in conducting quality clinical trials. Randomization and blinding though were seen to be increasingly done, but majority of the articles only mentioned their use without describing the procedure of how randomization and blinding were actually done. For other prospective studies, detailed results can be seen in S4–S6 Tables.

### Statistical Analysis

**Fig 1:**
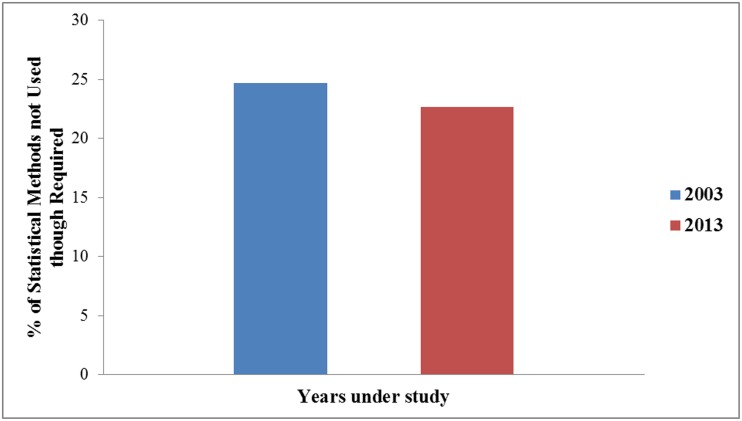
Percentage of statistical methods not used when required by year. This figure shows the percentage of articles which did not use any statistical methods even when required for the years 2003 and 2013.

**Fig 2:**
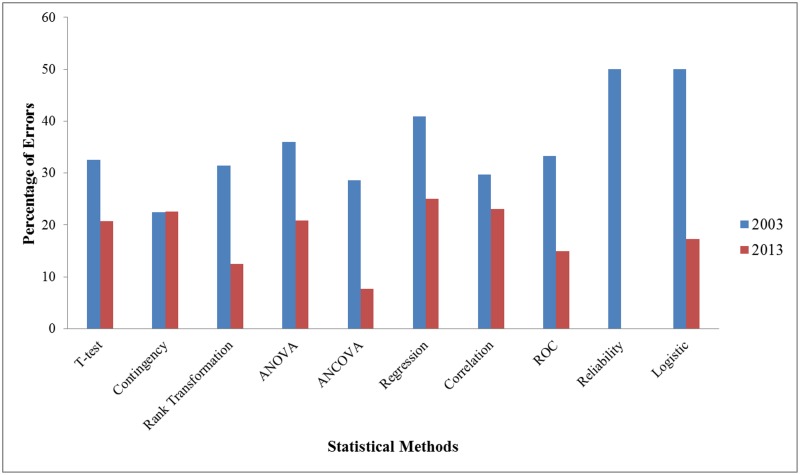
Percentage of errors in major statistical methods. This figure shows the percentage of errors present in major statistical methods in the years 2003 and 2013.

As in S4 Table, articles using statistical methods had increased markedly in (χ^2^ = 26.96, *Φ =* 0.14, *p*<0.0001) from 42.5% (250/588) to 56.7% (439/774). In 2003, 59.01% (347/588) articles had no statistical analyses, in which 13.4% (79/588) needed statistical analyses but were omitted. In 2013, 51.03% (395/774) articles had no statistical analyses, in which 14.34% (111/774) needed but statistical analyses were omitted (Fig 1). The most used statistical methods remained the simple tests (i.e., t-tests, contingency tables and ANOVA). However, some more sophisticated statistical methods, such as repeated-measures ANOVA, logistic regression and survival analysis were used in 2013. The proportion of erroneous statistical analyses had not decreased at all (*χ* ^*2*^ = 0.592, *Φ* = 0.027, *p* = 0.4418), 25% (80/320) in 2003 compared to 22.6% (111/490) in 2013. Statistical methods which had been misused frequently were not only seen in more complex tests but also observed in these simple tests. Compared with 2003, the error proportions of t-test were seen to decrease (t-test: 44.80% (26/58) compared to 24.5% (39/159), *χ* ^*2*^ = 8.346, *Φ* = 0.21, *p* = 0.0039) and for regression analyses (69.2% (9/13) in 2003 as compared to 33.33% (15/45) in 2013, *χ* ^*2*^ = 5.358, *Φ =* 0.31, *p* = 0.0206) but not for contingency tables (28.9% (24/83) compared to 27.9% (52/186), *χ* ^*2*^ = 0.026, *Φ =* 0.0098, *p* = 0.8719) (Fig 2). The most common mistakes for these methods were using multiple t-tests for multiple group comparisons, no significant level adjustment for multiple comparisons in association analysis (contingency tables), ignoring or misusing the method of multiple pair-wise comparisons in ANOVA, not reporting regression equation, not mentioning the beta-coefficients and not mentioning whether the basic assumptions were met. A complete summary of statistical methods can be seen in S7 and S8 Tables.

### Results, interpretation and presentation

Only study design, presentation and interpretation of results were seen to be improved, but serious errors/defects have remained (S9 Table). Inappropriate presentation of statistical results was the most common defect seen in both the observed years. Using arbitrary *p* thresholds (like *p*<0.01) instead of reporting exact p-values, reporting p value without test statistics, insufficient (or inappropriate) description of methods and *p* values without confidence intervals were most common of the errors present. The overall proportion of inappropriate presentation of results decreased significantly (*χ* ^*2*^ = 24.477, *Φ =* 0.178, *p*<0.0001), 82.2% (263/320) in 2003 compared to 66.3% (325/490) in 2013; Using arbitrary p thresholds instead of reporting exact p values had not decreased (*χ* ^*2*^ = 1.05, *Φ =* 0.036, *p* = 0.3055) from 2013 [33.7% (165/490)] as compared to [37.1%(119/320)] in 2003; reporting p value without test statistics had also not decreased [2003: 56.5% (181/320, χ^2^ = 1.313, *Φ =* 0.04, *p* = 0.2519); 2013: 60.6% (297/490)] inappropriate description of statistical methods was also down to 66.3% (325/490, *χ* ^*2*^ = 24.477, *Φ =* 0.178, *p*<0.0001). Also, the proportion of inappropriate interpretation of results was seen to be decreased (*χ* ^*2*^ = 25.616, *Φ =* 0.177, *p*<0.0001), 32.5% (104/320) compared to 17.1% (84/490).

## Discussion

Currently, RCT’s (Randomized clinical trial) is considered as the ‘‘gold standard” for evidence synthesis in clinical trials. But surprisingly, even in 2013, after 10 years’ of steady progress, randomized clinical trials published in Indian medical journals remained quite low (around 5%). Despite the publication of CONSORT and the widespread adoption in Western journals the quality of study design and statistical analyses of the RCT’s merely improved marginally. We hypothesize many reasons for this. First, Indian medical researchers and clinicians, in general, do not have a solid training in study designs. Very few medical colleges in India have attached statistics departments attached to them. Second, few of the better quality research papers have mostly been published in English in international journals. Along the lines of International Clinical Trials registry, India too established a similar registry for clinical trials. “*The Clinical Trials Registry—India (CTRI)*, *set up at the National Institute of Medical Statistics*, *ICMR*, *New Delhi is a free and online system for registration all clinical trials being conducted in India (www.ctri.nic.in). Registration of clinical trials in the CTRI is now mandatory*, *as per notification of the Drugs Controller General (India)*. [37]” We hence strongly endorse this requirement for full implementation of the clinical trial registration system to promote the quality of randomized clinical trials in India. Although a significant increase was seen in the rates of registration of clinical trials, the proportion of such trials remained low. We found that in 2013 study design types were unchanged since 2003 Retrospective studies still remain the most published one. Prospective clinical research, which includes randomized clinical trials and non- randomized clinical trials, only accounted for 12.3% in 2013. McDermott et al [30] in 1991 found that 35.0% papers published in JAMA, The Lancet and New England Journal of Medicine were clinical trials. This is seen in sharp contrast to the Indian condition even after 20 plus years later. Medical researchers should take the advantage of India’s large population, heavy cash investment in research by Indian Council of Medical Research (ICMR), Department of Biotechnology (DBT) and other organizations like Public Health Foundation of India (PHFI), broad disease spectrum found here (both lifestyle based and infectious), and quite a low cost to conduct high quality randomized clinical trials. Health policy-makers should encourage medical research by conducting more randomized clinical trials [36] with relevant guidance to the researchers to pay more attention to quality than quantity of their publications.

We noticed a massive increase in the use of rank based nonparametric test in 2013, which indicates that more attention are being paid to the assumptions of parametric test. This progress is mainly attributable to the emphasis of statistical education among medical postgraduates in India. In the author’s own university (Manipal University) every clinician-in-training is compulsorily given a statistics course both theoretical and practical using various statistical softwares like SPSS or R. Serious problems have remained nonetheless. Simple methods like t-tests, contingency tables, and ANOVA are seen to be used incorrectly. Regarding presentation of statistical results, even prestigious journals like Nature and BMJ were found to have a defect proportion of 38.0% and 25.0%, respectively [31–32]. Using arbitrary *p* thresholds (like p<0.01) instead of reporting exact p-values, reporting p value without test statistics, insufficient (or inappropriate) description of methods and *p* values without confidence intervals were most common of the error/defects observed in this study. Precise p-value and test statistic should be provided at the same time [17, 31]. We emphasize that, medical colleges should start teaching of the basic statistical concepts in a major way and hence strengthen statistical understanding among medical students. Also, in hospitals, a continuous education program in biostatistics should be encouraged among clinicians. According to the international practices followed by prestigious journals like JAMA, BMJ etc., editorial board should have qualified statisticians who should have a major say in checking the statistical quality of published papers.

The journals selected in this present study covered the important clinical fields and represented the top academic level of India based on impact factors released by Thomson Reuters in 2013. It should be noted, however, that high quality Indian research is regularly published in high-level international (e.g. American and European) journals. But in this study, we have not included such papers, which is a potential limitation. Also, another major limitation of this study is that since it’s primarily based on Indian medical journals hence, no generalization is possible with respect to other external, international journals.

In summary, this study indicates that Indian medical research seems to have made some progress regarding study design defects, but there remains ample room for improvement regarding statistical analyses. Most published studies continue to use a retrospective clinical design, with randomized clinical trials being quite rare. Those RCTs that are published often have serious methodological problems, including absence of sample size estimation and power calculations, as well as failure in (or in the reporting of) randomization. We therefore urgently recommend the full implementation of the CONSORT statement and of the registration system for clinical trials. It seems that medical research published in Indian journals still has much room for improvement, when compared to Western journals, not only in clinical knowledge, but also in study design and statistical methodology.

## Supporting Information

S1 Appendix Checklist. (DOCX)

S1 Table Descriptive data on 10 leading Indian medical journals (Based on Impact Factor) in 2003 and 2013. (DOCX)

S2 Table Data on study designs and the articles which used statistical analyses. (DOCX)

S3 Table Error/Defects in randomized clinical trial design. (DOCX)

S4 Table Error/Defects in cross-sectional study design. (DOCX)

S5 Table Error/Defects in cohort study design. (DOCX)

S6 Table Error/Defects in case-control study design. (DOCX)

S7 Table Descriptive summary about statistical methods used. (DOCX)

S8 Table Error/Defects in statistical methods—I (with χ2 and Φ values). (DOCX)

S9 Table Inappropriate presentation and/or interpretation of results. (DOCX)

S10 Table Errors in statistical methods—II (with Confidence Intervals). (DOCX)
