# Supplementary material for: Research Design and Statistical Methods in Indian Medical Journals: A Retrospective Survey
Source: PLoS One. 2015 Apr 9;10(4):e0121268. doi: 10.1371/journal.pone.0121268 (PMC4391869; doi:10.1371/journal.pone.0121268)
Supplement: S1 Appendix — (DOCX) [file pone.0121268.s001.docx]

| ID | Journal | Article |
| --- | --- | --- |

**Table 1: Errors/Defects in study design of Observational research**

| Study Design | Applied | Misused | Types of error |
| --- | --- | --- | --- |
| Case Report | (YES/NO) | (YES/NO) |  |
| Cross-Sectional Study | (YES/NO) | (YES/NO) | 1. No Sampling when needed (YES/NO) 2. Inappropriate sampling method or procedure (YES/NO) 3. No details of sampling procedure (YES/NO) 4. No description of any efforts to address potential sources of bias (YES/NO) 5. Eligibility criteria, and the sources and methods of selection of participants were NOT mentioned (YES/NO) 6. All outcomes, exposures, predictors, potential confounders, and effect modifiers are NOT described (YES/NO) 7. No sample size estimating step (YES/NO) 8. No analysis for non-response samples(YES/NO) |
| Cohort Study | (YES/NO) | (YES/NO) | 1. NO Sampling when needed (YES/NO) 2. Inappropriate sampling method or procedure(YES/NO) 3. NO mention of eligibility criteria, and the sources and methods of selection of participants (YES/NO) 4. Follow-up procedures are not mentioned (YES/NO) 5. For matched studies,the matching criteria and number of exposed and unexposed were NOT provided (YES/NO) 6. NO details of sampling procedure (YES/NO) 7. NO sample size estimating step (YES/NO) 8. NO inclusion and exclusion criteria (YES/NO) 9. Inappropriate non-exposed group (YES/NO) 10. NO analysis for withdrawals (YES/NO) |
| Case-Control Study | (YES/NO) | (YES/NO) | 1. No Sampling when needed (YES/NO) 2. Inappropriate sampling method or procedure(YES/NO) 3. No description of the study population (YES/NO) 4. No eligibility criteria, and the sources and methods of case ascertainment and control selection are provided (YES/NO) 5. No rationale for the choice of cases and controls (YES/NO) 6. For matched studies, no matching criteria and the number of controls per case were provided (YES/NO) 7. No description of any efforts to address potential sources of bias(YES/NO) 8. No definition of outcomes, exposures, predictors, potential confounders, and effect modifiers (YES/NO) 9. No details of sampling procedure (YES/NO) 10. No sample size estimating step (YES/NO) 11. No inclusion and exclusion criteria (YES/NO) 12. Inappropriate control group (YES/NO) |
| Diagnostic Test | (YES/NO) | (YES/NO) | 1. No sample size estimating step (YES/NO) 2. No control group or inappropriate control group (YES/NO) 3. No description of the reference group and its rationale (YES/NO) 4. No description of the study population (YES/NO) 5. No method for calculating or comparing measures of diagnostic accuracy, and the statistical methods used to quantify uncertainty (e.g. 95% confidence intervals) (YES/NO) 6. No description of whether or not the readers of the index tests and reference standard were blind (masked) to the results of the other test and describe any other clinical information available to the readers (YES/NO) 7. No report on the number of participants satisfying the criteria for inclusion that did or did not undergo the index tests and/or the reference standard; and no description of why participants failed to receive either test (a flow diagram is strongly recommended) (YES/NO) |

**Table 2: Defects in study design of intervention research**

| Study Design | Applied | Misused | Types of Error |
| --- | --- | --- | --- |
| RCT | (YES/NO) | (YES/NO) | 1. Unclear study aim and hypothesis (YES/NO) 2. Unclear primary outcome measures (YES/NO) 3. No sample size estimating step (YES/NO) 4. No inclusion and exclusion criteria (YES/NO) 5. No statement of intervention for each group or unclear (YES/NO) 6. Failure to use or report randomization (YES/NO) 7. No report of blindness when needed (YES/NO) 8. No analysis for withdrawals (YES/NO) 9. Was the RCT registered on clinical trial registration platform (YES/NO) |
| Non-RCT | (YES/NO) | (YES/NO) | 1. Unclear study aim and hypothesis (YES/NO) 2. Unclear primary outcome measures (YES/NO) 3. No sample size estimating step (YES/NO) 4. No inclusion and exclusion criteria (YES/NO) 5. No statement of intervention for each group or unclear (YES/NO) 6. Failure to use or report randomization (YES/NO) 7. No report of blindness when needed (YES/NO) 8. No analysis for withdrawals (YES/NO) |
| Basic Science Study | (YES/NO) | (YES/NO) | 1. No sample size estimating step (YES/NO) 2. Failure to report the test power (YES/NO) 3. No statement of intervention for each group or unclear (YES/NO) 4. Failure to use or report randomization (YES/NO) 5. No control group (YES/NO) 6. Use of inappropriate control group (YES/NO) |

Does the research need statistical analysis?

| if YES | did it fail to use | NO name of the statistical software package used in the analysis |
| --- | --- | --- |

**Table 3: Statistical content and errors/defects in original articles**

| Statistical Methods | Applied | Misused | Types of Error |
| --- | --- | --- | --- |
| Statistical description | (YES/NO) | (YES/NO) | 1. No report of numbers-especially measurements-without an appropriate degree of precision (YES/NO) 2. Total sample and group sizes for each analysis are not reported (YES/NO) 3. Numerators and denominators for all percentages are not mentioned (YES/NO) 4. Data that are approximately normally distributed with means and standard deviations (SD) are NOT summarized (YES/NO) 5. Data that are not normally distributed are NOT summarized with medians and inter-percentile ranges, ranges, or both (YES/NO) 6. Standard error of the mean (SE) WAS USED to indicate the variability of a data set instead of standard deviations, inter-percentile ranges, or ranges (The SE is an inferential statistic-it is about a 68% confidence interval-not a descriptive statistic.) (YES/NO) 7. Data in tables or figures are NOT displayed correctly. Tables present exact values, and figures provide an overall assessment of the data (YES/NO) |
| Risk, rates and ratios | (YES/NO) | (YES/NO) | 1. No identification of the type of rate (e.g., incidence rates; survival rates), ratio (e.g., odds ratios; hazards ratios), or risk (e.g., absolute risks; relative risk differences), being reported (YES/NO) 2. No identification of the quantities represented in the numerator and denominator (e.g., the number of men with prostate cancer divided by the number of men in whom prostate cancer can occur) (YES/NO) 3. No identification of the time period over with each rate applies (YES/NO) 4. No identification of any unit of population (that is, the unit multiplier: e.g., x 100; x 10,000) associated with the rate (YES/NO) 5. No reporting a measure of precision (a confidence interval) for estimated risks, rates, and ratios (YES/NO) 6. No hypothesis is described which is being tested (YES/NO) 7. No identification of the variables in the analysis and either no/partial summarization of the data for each variable with the appropriate descriptive statistics (YES/NO) 8. No identification of minimum difference considered to be clinically important (YES/NO) 9. For equivalence and non-inferiority studies, NO report of the largest difference between groups that will still be accepted as indicating biological equivalence (the equivalence margin) (YES/NO) 10. No identification of the name of the test used in the analysis (YES/NO) 11. No report of whether the test was one- or two-tailed (justify the use of one-tailed tests) and for paired or independent samples (YES/NO) 12. No confirmation that the assumptions of the test were met by the data (YES/NO) 13. No report of the alpha level (e.g., 0.05) that defines statistical significance (YES/NO) 14. At least for primary outcomes, such as differences or agreement between groups, diagnostic sensitivity, and slopes of regression lines, NO REPORT of a measure of precision, such as the 95% confidence interval (YES/NO) 15. Standard error of the mean (SE) is NOT used to indicate the precision of an estimate. The SE is essentially a 68% confidence coefficient: use the 95% confidence coefficient instead (YES/NO) 16. P values are not reported as equalities when possible and to one or two decimal places (YES/NO) 17. Report NOT SIGNIFICANT; instead of the actual P value. The smallest P value that need be reported is P is less than 0.001 (YES/NO) 18. NO report of whether and how any adjustments were made for multiple statistical comparisons (YES/NO) |
| t-test | (YES/NO) | (YES/NO) | 1. Using multiple t-test for multiple group comparison (YES/NO) 2. Using paired t-test for unpaired data or vice versa (YES/NO) 3. Using t-test under nonparametric setting (YES/NO) 4. Using t-test without considering the baseline (YES/NO) |
| Association analyses | (YES/NO) | (YES/NO) | 1. Association of interest is NOT described (YES/NO) 2. Did NOT identify the variables used (YES/NO) 3. Did NOT summarize each with descriptive statistics (YES/NO) 4. Test of association used is NOT identified (YES/NO) 5. Did NOT indicate whether the test was one- or two-tailed (YES/NO) 6. NO justification for the use of one-tailed tests (YES/NO) 7. For tests of association (e.g., a chi-square test), NO report of the P value of the test is given (because association is defined as a statistically significant result) (YES/NO) 8. Association is described as low, moderate, or high when the ranges for these categories have NOT been defined (YES/NO) 9. Biological implications or realities are NOT given for categories of association above (YES/NO) 10. For primary comparisons, full contingency table is NOT given for the analysis (YES/NO) 11. No continuity correction or Fisher exact test if needed (YES/NO) 12. No significant level adjustment for multiple comparison (YES/NO) 13. Misusing Chi-square test for paired fourfold table (YES/NO) 14. Misusing Chi-square test for paired fourfold table (YES/NO) 15. Ignorance of stratification factors (YES/NO) 16. Using p value of Chi-square test to describe the correlation of two variables instead of contingency coefficient (YES/NO) |
| Rank transformation nonparametric test | (YES/NO) | (YES/NO) | 1. Using multiple pair-wise comparison for multiple group comparison (YES/NO) 2. Using wrong type of rank sum test for different study types (YES/NO) |
| ANOVA | (YES/NO) | (YES/NO) | 1. Completely random ANOVA was used to analyze multi-factor ANOVA (YES/NO) 2. Ignoring the setting of ANOVA for completely random design data ANOVA (YES/NO) 3. No multiple pair-wise comparison of ANOVA when needed (YES/NO) 4. Misusing the method of multiple pair-wise comparison of ANOVA (YES/NO) 5. Using ANOVA to analyze repeated-measures data (YES/NO) |
| ANCOVA/ANOVA/Repeated Measures | (YES/NO) | (YES/NO) | 1. NO description of the purpose of the analysis (YES/NO) 2. Variables used in the analysis and summary of each with descriptive statistics is NOT provided (YES/NO) 3. NO confirmation that the assumptions of the analysis were met (YES/NO) 4. NO description of how any outlying values were treated in the analysis (YES/NO) 5. NO report of how any missing data were treated in the analyses (YES/NO) 6. NO specification whether the explanatory variables were tested for interaction, and if so how these interactions were treated (YES/NO) 7. If appropriate, in a table, P value for each explanatory variable, the test statistics and, where applicable, the degrees of freedom for the analysis was NOT reported (YES/NO) 8. NO assessment of the goodness-of-fit of the model to the data, such as R2 was given (YES/NO) |
| Regression Analysis | (YES/NO) | (YES/NO) | 1. NO description of the purpose of the analysis (YES/NO) 2. Variables used in the analysis and summary of each with descriptive statistics is NOT provided (YES/NO) 3. NO description of assumptions of the analysis (YES/NO) 4. NO description of how any outlying values were treated in the analysis (YES/NO) 5. NO report of how any missing data were treated in the analyses (YES/NO) 6. For either simple or multiple (multivariable) regression analyses, regression equation was not reported (YES/NO) 7. For multiple regression analyses: 1) ralpha level used in the univariate analysis is NOT reported; (YES/NO) 8. For multiple regression analyses: 2) report whether the variables were assessed for a) colinearity and b) interaction; (YES/NO) 9. For multiple regression analyses: 3) variable selection process by which the final model was developed (e.g., forward-stepwise; best subset) was NOT described (YES/NO) 10. NO reporting of the regression coefficients (beta weights) of each explanatory variable and the associated confidence intervals and P values, preferably in a table was given. (YES/NO) 11. Measure of the model's "goodness-of-fit" to the data (the coefficient of determination, r2, for simple regression and the coefficient of multiple (YES/NO)determination, R2, for multiple regression was NOT provided (YES/NO) 12. NO model validation procedure was given (YES/NO) 13. For primary comparisons analyzed with simple linear regression analysis,results was NOT presented graphically, in a scatter plot showing the regression line and its confidence bounds. (YES/NO) 14. Regression line (or the interpretation of the analysis) beyond the minimum and maximum values of the data WAS EXTENDED IN THE PLOT. (YES/NO) |
| Survival Analysis | (YES/NO) | (YES/NO) | 1. NO description of purpose of the analysis (YES/NO) 2. Dates or events that mark the beginning and the end of the time period analyzed are NOT reported (YES/NO) 3. Circumstances under which data were censored IS NOT mentioned (YES/NO) 4. Statistical methods used to estimate the survival rate is NOT mentioned (YES/NO) 5. NO confirmation that the assumptions of survival analysis were met. (YES/NO) 6. For each group, NO estimatation of survival probability at appropriate follow-up times, with confidence intervals, and the number of participants at risk for death at each time was given. It is often more helpful to plot the cumulative probability of not surviving, especially when events are not common (YES/NO) 7. NO reporting of median survival times, with confidence intervals (YES/NO) 8. NO graph (e.g., a Kaplan-Meier plot) or table is provided (YES/NO) 9. NO specification of the statistical methods used to compare two or more survival curves is provided (YES/NO) 10. When comparing two or more survival curves with hypothesis tests, no P value of the comparison is provided (YES/NO) 11. NO Regression model was specified (YES/NO) 12. NO reporting of measure of risk (e.g., a hazard ratio) for each explanatory variable, with a confidence interval was provided (YES/NO) |
| Clustering Analysis | (YES/NO) | (YES/NO) |  |
| Discriminant Analysis | (YES/NO) | (YES/NO) |  |
| PCA & Factor Analysis | (YES/NO) | (YES/NO) |  |
| Correlation analysis | (YES/NO) | (YES/NO) | 1. NO description of purpose of the analysis (YES/NO) 2. NO summary of each variable without the appropriate descriptive statistics. (YES/NO) 3. NO identification of correlation coefficient used in the analysis (e.g., Pearson, Spearman). (YES/NO) 4. NO confirmation that the assumptions of the analysis were met (YES/NO) 5. NO report the alpha level (e.g., 0.05) that indicates whether the correlation coefficient is statistically significant (YES/NO) 6. NO report of value of the correlation coefficient. Do not describe correlation as low, moderate, or high unless the ranges for these categories have been defined. Even then, consider the wisdom of using these categories given their biological implications or realities. (YES/NO) 7. For primary comparisons, the (95%) confidence interval for the correlation coefficient was NOT reported, whether or not it is statistically significant (YES/NO) 8. For primary comparisons, scatter plot was NOT presented. The sample size, correlation coefficient (with its confidence interval), and P value HAS NOT been included in the data field (YES/NO) |
| Meta analysis | (YES/NO) | (YES/NO) |  |
| ROC | (YES/NO) | (YES/NO) |  |
| Reliability & Validity Analysis | (YES/NO) | (YES/NO) |  |
| Bayesian Analysis | (YES/NO) | (YES/NO) | 1. NO specification of the pre-trial probabilities ("priors"). (YES/NO) 2. NO explanation of how the priors were selected (YES/NO) 3. NO description of the statistical model used (YES/NO) 4. NO description of the techniques used in the analysis (YES/NO) 5. NO identification of the statistical software program used in the analysis (YES/NO) 6. NO summary of the posterior distribution with a measure of central tendency and a credibility interval (YES/NO) 7. NO assessment of sensitivity of the analysis to different priors (YES/NO) |
| Simple Linear Regression | (YES/NO) | (YES/NO) |  |
| Multiple Linear Regression | (YES/NO) | (YES/NO) |  |
| Multivariate Regression | (YES/NO) | (YES/NO) |  |
| Logistic Regression | (YES/NO) | (YES/NO) |  |

**Table 4: Inappropriate presentation & Interpretation of results**

| Result presentation & Interpretation | (YES/NO) |
| --- | --- |
| Unspecified statistical methods | (YES/NO) |
| Insufficient or inappropriate description of methods | (YES/NO) |
| Reporting p value without test statistics | (YES/NO) |
| p values without confidence intervals | (YES/NO) |
| Using mean±SD to describe non-normal data | (YES/NO) |
| Using proportion instead of rate | (YES/NO) |
| Using arbitrary p thresholds instead of reporting exact p values | (YES/NO) |
| Incorrect use of table | (YES/NO) |
| Incorrect use of chart and figure | (YES/NO) |
| Ignoring baselines of two groups | (YES/NO) |
| p less than a ,the smaller the P value, the greater the difference between groups | (YES/NO) |
| p less than a ,reporting no difference between groups | (YES/NO) |
| p greater than a ,reporting difference between groups | (YES/NO) |
