# Supplementary material for: Research Design and Statistical Methods in Indian Medical Journals: A Retrospective Survey
Source: PLoS One. 2015 Apr 9;10(4):e0121268. doi: 10.1371/journal.pone.0121268 (PMC4391869; doi:10.1371/journal.pone.0121268)
Supplement: S1 Table — (DOCX) [file pone.0121268.s002.docx]

| **Table S1. Descriptive data on 10 leading Indian medical journals (Based on Impact Factor)** | | | | | |
| --- | --- | --- | --- | --- | --- |
| Journals | 2003 | |  | 2013 | |
|  | # Issues | # Articles |  | # Issues | # Articles |
| Indian Journal of Medical Research | 12 | 66 |  | 12 | 155 |
| Indian Journal of Medical Sciences | 12 | 62 |  | 12 | 22 |
| Indian Journal of Otolaryngology & Head and Neck Surgery | 4 | 91 |  | 4 | 212 |
| The Indian Journal of Chest Diseases & Allied Sciences | 12 | 136 |  | 12 | 40 |
| Journal of Postgraduate Medicine | 4 | 43 |  | 4 | 27 |
| Indian Journal of Dermatology, Venereology, Leprology | 6 | 71 |  | 6 | 63 |
| Indian Journal of Cancer | 3 | 15 |  | 4 | 65 |
| Neurology India | 4 | 52 |  | 4 | 63 |
| Indian Pediatrics | 12 | 174 |  | 12 | 233 |
| Indian Journal of Ophthalmology | 4 | 27 |  | 12 | 53 |
| Total | 73 | 737 |  | 82 | 933 |
