# Supplementary material for: Research Design and Statistical Methods in Indian Medical Journals: A Retrospective Survey
Source: PLoS One. 2015 Apr 9;10(4):e0121268. doi: 10.1371/journal.pone.0121268 (PMC4391869; doi:10.1371/journal.pone.0121268)
Supplement: S2 Table — (DOCX) [file pone.0121268.s003.docx]

| **Table S2. Data on study designs and the articles which used statistical analyses** | | | | | | | | |
| --- | --- | --- | --- | --- | --- | --- | --- | --- |
| Study Design | 2003 | | | 2013 | | |  |  |
|  | # Articles | # Articles used statistical analyses  n (%)* | # Articles had design defects  n (%) | # Articles | # Articles used statistical analyses  n (%)* | # Articles had design defects  n (%) | |  |
| Systematic Review | 1 | 1(100%) | 0(0%) | 3 | 2(66.67%) | 1(33.3%) |  |  |
| Randomized clinical trial | 43 | 38(88.4%) | 41(95.3%) | 41 | 35(85.4%) | 30(73.2%) |  |  |
| Non- randomized clinical trial | 27 | 10(37%) | 23(85.2%) | 54 | 35(64.8%) | 26(48.1%) |  |  |
| Cohort Study | 12 | 5(41.6%) | 12(100%) | 50 | 45(90%) | 27(54%) |  |  |
| Case-Control Study | 31 | 19(61.3%) | 21(67.7%) | 60 | 52(86.7%) | 23(38.3%) |  |  |
| Cross-Sectional Study | 177 | 128(72.3%) | 101(57.1%) | 272 | 218(80.1%) | 92(33.8%) |  |  |
| Case Study or Case Series Study | 209 | 0(0%) | 3(1.45%) | 205 | 0(0%) | 6(2.9%) |  |  |
| Diagnostic Test | 36 | 28(77.78%) | 21(58.3%) | 18 | 15(83.3%) | 9(50%) |  |  |
| Basic-science study** | 52 | 21(40.4%) | 21(59.6%) | 62 | 37(59.67%) | 15(24.2%) |  |  |
| Total | 588 | 250(42.5%) | 243(41.3%) | 774 | 439(56.7%) | 237(30.6%) |  |  |

*Number of articles which used statistical analyses n (%): Here, n is the number of articles which uses statistical methods, % = n/total number of articles with a particular kind of study design×100%.

**Basic science study would also include all preclinical animal and laboratory both in vivo and in vitro studies.
