# Supplementary material for: Research Design and Statistical Methods in Indian Medical Journals: A Retrospective Survey
Source: PLoS One. 2015 Apr 9;10(4):e0121268. doi: 10.1371/journal.pone.0121268 (PMC4391869; doi:10.1371/journal.pone.0121268)
Supplement: S3 Table — (DOCX) [file pone.0121268.s004.docx]

| **Table S3. Error/Defects in RCT (Randomised clinical trial) design** | | |
| --- | --- | --- |
| Error/Defect in randomized clinical trial | 2003  # articles  n (%)  (N=43) | 2013  # articles  n (%)  (N=41) |
| Unclear study aim and hypothesis | 14(29.16%) | 3(6.67%) |
| Unclear primary outcome measures | 23(47.91%0 | 5(11.11%) |
| No sample size estimating step | 39 (91.67%) | 31(28.89%) |
| No inclusion and exclusion criteria | 28(58.34%) | 7(15.56%) |
| No statement of intervention for each group or unclear | 7(14.58%) | 1(2.23%) |
| Failure to use or report randomisation | 29(60.41%) | 9(20%) |
| No report of blindness when needed | 29(60.41%) | 15(33.34%) |
| No analysis for withdrawls | 18(37.5%) | 15(33.34%) |
| Was the RCT registered on clinical trial registration platform | 16(33.34%) | 24(53.34%) |

Here, N= total number of articles with RCT (randomized clinical trial) design
