# Supplementary material for: Research Design and Statistical Methods in Indian Medical Journals: A Retrospective Survey
Source: PLoS One. 2015 Apr 9;10(4):e0121268. doi: 10.1371/journal.pone.0121268 (PMC4391869; doi:10.1371/journal.pone.0121268)
Supplement: S4 Table — (DOCX) [file pone.0121268.s005.docx]

| **Table S4. Error/Defects in Cross-Sectional Studies design** | | |
| --- | --- | --- |
| Error/Defect in cross-sectional Studies | 2003  # articles  n (%)  (N=201) | 2013  # articles  n (%)  (N=317) |
| No Sampling when needed | 54(26.86%) | 113(35.64%) |
| Inappropriate sampling method or procedure | 62(30.84%) | 56(17.66%) |
| No details of sampling procedure | 86(42.78%) | 226(71.29%) |
| No description of any efforts to address potential sources of bias | 134(66.67%) | 173(54.57%) |
| Eligibility criteria, and the sources and methods of selection of participants were NOT mentioned | 107(53.23%) | 115(36.27%) |
| All outcomes, exposures, predictors, potential confounders, and effect modifiers are NOT described | 123(69.19%) | 114(35.96%) |
| No sample size estimating step | 179(89.05%) | 277(87.38%) |
| No analysis for non-response samples | 39(19.40%) | 80(25.23%) |

Here, N= total number of articles with cross-sectional studies design
