# Supplementary material for: Research Design and Statistical Methods in Indian Medical Journals: A Retrospective Survey
Source: PLoS One. 2015 Apr 9;10(4):e0121268. doi: 10.1371/journal.pone.0121268 (PMC4391869; doi:10.1371/journal.pone.0121268)
Supplement: S5 Table — (DOCX) [file pone.0121268.s006.docx]

| **Table S5. Error/Defects in cohort study design** | | |
| --- | --- | --- |
| Error/Defect in cohort study | 2003  # articles  n (%)  (N=17) | 2013  # articles  n (%)  (N=67) |
| No Sampling when needed | 8(47.05%) | 19(28.35%) |
| Inappropriate sampling method or procedure | 10(58.82%) | 1928.35%) |
| No mention of eligibility criteria, and the sources and methods of selection of participants | 13(76.47%) | 23(34.32%) |
| Follow-up procedures are not mentioned | 8(47.05%) | 21(31.34%) |
| For matched studies, the matching criteria and number of exposed and unexposed were NOT provided | 5(29.41%) | 3(4.47%) |
| No details of sampling procedure | 12(70.58%) | 47(70.14%) |
| No sample size estimating step | 15(88.23%) | 59(88.05%) |
| No inclusion and exclusion criteria | 13(76.47%) | 13(19.40%) |
| Inapproriate non-exposed group | 6(35.29%) | 8(11.94%) |
| No analysis for withdrawls | 2(11.76%) | 26(38.80%) |

Here, N= total number of articles with cohort study design
