# Supplementary material for: Research Design and Statistical Methods in Indian Medical Journals: A Retrospective Survey
Source: PLoS One. 2015 Apr 9;10(4):e0121268. doi: 10.1371/journal.pone.0121268 (PMC4391869; doi:10.1371/journal.pone.0121268)
Supplement: S6 Table — (DOCX) [file pone.0121268.s007.docx]

| **Table S6. Error/Defects in case-control study design** | | |
| --- | --- | --- |
| Error/Defect in case-control study | 2003  # articles  n (%)  (N=41) | 2013  # articles  n (%)  (N=73) |
| No Sampling when needed | 22(53.65%) | 13(17.81%) |
| Inappropriate sampling method or procedure | 23(56.09%) | 13(17.81%) |
| No description of the study population | 17(41.46%) | 32(43.84%) |
| No eligibility criteria, and the sources and methods of case ascertainment and control selection are provided | 30(73.17%) | 23(31.51%) |
| No rationale for the choice of cases and controls | 20(48.78%) | 13(17.81%) |
| For matched studies, no matching criteria and the number of controls per case were provided | 4(9.75%) | 1(1.37%) |
| No description of any efforts to address potential sources of bias | 34(82.92%) | 39(53.42%) |
| No definition of outcomes, exposures, predictors, potential confounders, and effect modifiers | 25(60.98%) | 14(19.18%) |
| No details of sampling procedure | 23(56.09%) | 51(69.86%) |
| No sample size estimating step | 41(100%) | 66(90.41%) |
| No inclusion and exclusion criteria | 34(82.92%) | 6(8.22%) |
| Inappropriate control group | 11(26.82%) | 9(12.33%) |

Here, N= total number of articles with case-control study design
