# Supplementary material for: Research Design and Statistical Methods in Indian Medical Journals: A Retrospective Survey
Source: PLoS One. 2015 Apr 9;10(4):e0121268. doi: 10.1371/journal.pone.0121268 (PMC4391869; doi:10.1371/journal.pone.0121268)
Supplement: S7 Table — (DOCX) [file pone.0121268.s008.docx]

| **Table S7. Descriptive summary about statistical methods used** | | | | | |
| --- | --- | --- | --- | --- | --- |
| Statistical methods | 2003 (N=588) | |  | 2013 (N=774) | |
|  | All articles  n (%) | Incorrect use  n (%) |  | All articles  n (%) | Incorrect use  n (%) |
| No statistical methods or description* | 347(59.01%) | - |  | 395(51%) | - |
| Articles which uses statistical methods | 241(40.9%) | 99(41.1%) |  | 379(48.9%) | 179(36.5%) |
| t-test | 58(9.8%) | 28(48.3%) |  | 159(20.5%) | 39(24.5%) |
| Contingency tables | 83(14.1%) | 24(28.9%) |  | 186(24.03%) | 52(27.9%) |
| Rank transformation nonparametric test | 24(4.08%) | 11(45.8%) |  | 86(11.1%) | 12(13.9%) |
| ANOVA | 16(2.7%) | 9(56.3%) |  | 64(8.2%) | 16(25%) |
| Repeated-measures analysis | 15(2.5%) | 6(40%) |  | 25(3.2%) | 15(60%) |
| Correlation Analysis | 26(4.4%) | 11(42.3%) |  | 50(6.5%) | 15(30%) |
| Simple linear regression | 2(0.3%) | 1(50%) |  | 7(0.9%) | 3(42.8%) |
| Multiple linear regression | 4(0.6%) | 4(100%) |  | 11(1.4%) | 3(27.2%) |
| Logistic regression | 3(0.5%) | 3(100%) |  | 43(5.56%) | 9(20.9%) |
| Survival analysis | 1(0.1%) | 0(0%) |  | 27(3.5%) | 10(37.03%) |
| Bayesian Analysis | 0(0%) | 0(0%) |  | 0(0%) | 0(0%) |
| ROC | 2(0.3%) | 1(50%) |  | 17(2.2%) | 3(17.7%) |
| Others** | 1(0.1%) | 1(100%) |  | 21(2.7%) | 2(9.5%) |

N=total articles (2003:588; 2013:774)

All articles n (%): percentage=n/N(total articles)×100%,

**Incorrect use n (%):** Here, n is the number of articles which used statistical methods incorrectly; **%** = n/ number of papers which use certain statistical methods×100%.

*Including articles do not need statistical methods and articles need statistical methods but omitted (2003:79; 2013: 111)

**These include clustering analysis (1 article), discriminant analysis (0), meta-analysis (7), Reliability and validity analysis (12) and PCA (1)
