# Supplementary material for: Research Design and Statistical Methods in Indian Medical Journals: A Retrospective Survey
Source: PLoS One. 2015 Apr 9;10(4):e0121268. doi: 10.1371/journal.pone.0121268 (PMC4391869; doi:10.1371/journal.pone.0121268)
Supplement: S8 Table — (DOCX) [file pone.0121268.s009.docx]

| **Types of Errors** | **Incorrect use in 2003**  **n(%)** | **Incorrect use in 2013**  **n(%)** | **Χ^2^** | **Effect Size**  **(Φ)** | **P-value** |
| --- | --- | --- | --- | --- | --- |
| **t-test** |  |  |  |  |  |
| 1. Using multiple t-test for multiple group comparison | 2(3.4%) | 12(7.5%) | - | 0.0737 | 0.3623 |
| 1. Using paired t-test for unpaired data or vice versa | 2(3.4%) | 0(0%) | - | 0.1596 | 0.0705 |
| 1. Using t-test under nonparametric setting | 9(15.5%) | 20(11.1%) | 0.317 | 0.0384 | 0.5734 |
| 1. Using t-test without considering the baseline | 3(5.2%) | 6(3.35%) | - | 0.0311 | 0.7034 |
| **Contingency Tables** |  |  |  |  |  |
| 1. Association of interest is NOT described | 70(84.3%) | 33(17.7%) | 107.718 | 0.6328 | <0.0001 |
| 1. Did NOT identify the variables used | 56(67.4%) | 40(21.5%) | 52.829 | 0.4432 | <0.0001 |
| 1. Did NOT summarize each with descriptive statistics | 33(39.7%) | 78(41.9%) | 0.112 | 0.0202 | 0.7377 |
| 1. Test of association used is NOT identified | 39(46.9%) | 33(17.7%) | 25.043 | 0.3051 | <0.0001 |
| 1. Did NOT indicate whether the test was one- or two-tailed | 78(94.9%) | 125(67.2%) | 22.215 | 0.2874 | <0.0001 |
| 1. NO justification for the use of one-tailed tests | 3(3.6%) | 26(13.9%) | 6.429 | 0.1544 | 0.0114 |
| 1. For tests of association (e.g., a chi-square test), no report of the P value of the test is given | 48(57.8%) | 26(13.9%) | 55.344 | 0.4536 | <0.0001 |
| 1. Association is described as low, moderate, or high when the ranges for these categories have not been defined | 0(0%) | 2(1.07%) | - | 0.0578 | 0.9880 |
| **Types of Errors** | **Incorrect use in 2003**  **n(%)** | **Incorrect use in 2013**  **n(%)** | **Χ^2^** | **Effect Size**  **(Φ)** | **P-value** |
| 1. For primary comparisons, full contingency table is NOT given for the analysis | 20(24.1%) | 85(45.7%) | 11.254 | 0.2045 | 0.0008 |
| 1. Using p value of Chi-square test to describe the correlation of two variables instead of contingency coefficient | 2(2.4%) | 8(4.3%) | - | 0.046 | 0.7286 |
| **Regression Analysis** |  |  |  |  |  |
| 1. NO description of assumptions of the analysis | 5(38.4%) | 15(32.6%) | 0.117 | 0.0455 | 0.7319 |
| 1. NO description of how any outlying values were treated in the analysis | 12(92.3%) | 32(69.6%) | - | 0.2064 | 0.1553 |
| 1. NO report of how any missing data were treated in the analyses | 13(100%) | 25(54.3%) | - | 0.39 | 0.0022 |
| 1. For either simple or multiple (multivariable) regression analyses, regression equation was not reported | 12(92.3%) | 19(41.3%) | - | 0.4187 | 0.0024 |
| 1. For multiple regression analyses: 3) variable selection process by which the final model was developed | 9(69.2%) | 11(23.9%) | - | 0.3928 | 0.0062 |
| 1. NO reporting of the regression coefficients (beta weights) of each explanatory variable and the associated confidence intervals | 9(69.2%) | 30(65.2%) | - | 0.0277 | 0.9875 |
| 1. Measure of the model's "goodness-of-fit" to the data | 8(61.5% | 40(86.7%) | 5.288 | 0.302 | 0.0215 |
| 1. NO model validation procedure was given | 10(76.9%) | 24(52.2%) | - | 0.1996 | 0.2017 |
| 1. For primary comparisons analyzed with simple linear regression analysis, results was NOT presented graphically | 9(69.2%) | 19(41.3%) | 2.947 | 0.2255 | 0.0861 |
| 1. Regression line (or the interpretation of the analysis) beyond the minimum and maximum values of the data was extended in the plot | 2(15.4%) | 7(15.2%) | - | 0.0557 | 0.972 |

Incorrect use of *n* (%): for each statistical method, *n* is the number of articles using this statistical methods incorrectly and the percentage = *n*/the number of papers using this statistical methods × 100%; for each error under certain statistical methods, *n* is the number of articles with this mistake and the percentage = *n*/ the number of papers using these statistical methods × 100%.

For cells with no value in chi-square column, the p-value was obtained via Fisher’s Test
