# Supplementary material for: Research Design and Statistical Methods in Indian Medical Journals: A Retrospective Survey
Source: PLoS One. 2015 Apr 9;10(4):e0121268. doi: 10.1371/journal.pone.0121268 (PMC4391869; doi:10.1371/journal.pone.0121268)
Supplement: S9 Table — (DOCX) [file pone.0121268.s010.docx]

| **Table S9. Inappropriate presentation and/or interpretation of results** | | |
| --- | --- | --- |
| Inappropriate presentation and/or interpretation of results | 2003 (N=320)  # articles, n (%)* | 2013 (N=490)  # articles, n (%)* |
| Using arbitrary *p* thresholds (like p<0.01) instead of reporting exact p-values | 119(37.2%) | 165(33.67%) |
| Reporting p value without test statistics | 181(56.6%) | 297(60.6%) |
| Insufficient (or inappropriate) description of methods | 263(82.2%) | 325(66.3%) |
| *p* values without confidence intervals | 171(53.4%) | 217(44.3%) |
| Unspecified statistical methods | 194(60.6%) | 294(60%) |
| Using mean±SD to describe non-normal data | 29(9.1%) | 59(12%) |
| Using proportion instead of rate | 6(1.8%) | 5(1.02%) |
| Incorrect use of table | 104(32.5%) | 84(17.1%) |
| Incorrect use of chart/figure | 11(3.4%) | 27 (5.5%) |
| Ignoring baselines of two groups | 21(6.5%) | 56(11.4%) |
| p<$\alpha$，the smaller the *p* value is, the greater the difference between groups is | 1(0.3%) | 5 (1.02%) |
| P<$\alpha$，reporting no difference between groups | 2(0.6%) | 1(0.2%) |
| p>$\alpha$，reporting difference between groups | 1(0.3%) | 0(0%) |

N=articles which needed statistical methods=articles used statistical analyses + articles need statistical methods but omitted (2003: 320=241+79; 2013: 490=379+111)

*n (%): percentage=n/N (articles which needed statistical methods) ×100%
