# Supplementary material for: Research Design and Statistical Methods in Indian Medical Journals: A Retrospective Survey
Source: PLoS One. 2015 Apr 9;10(4):e0121268. doi: 10.1371/journal.pone.0121268 (PMC4391869; doi:10.1371/journal.pone.0121268)
Supplement: S10 Table — (DOCX) [file pone.0121268.s011.docx]

| **S.No** | **TESTS** | **2003** | | **2013** | | **CI** | | **Difference in proportion** |
| --- | --- | --- | --- | --- | --- | --- | --- | --- |
|  |  | **kA** | **nA** | **kB** | **nB** | **Lower** | **Upper** |  |
| **1** | Statistical Analysis | 80 | 320 | 111 | 490 | -0.0374 | 0.0865 | 0.0235 |
| **2** | Using Statistical Test | 250 | 588 | 439 | 774 | 0.0875 | 0.1954 | 0.142 |
| **3** | Study Design Errors | 243 | 588 | 237 | 774 | 0.0546 | 0.1592 | 0.1071 |
| **4** | RCT’s # | 43 | 588 | 41 | 774 | -0.0067 | 0.0488 | 0.0202 |
| **5** | RCT’s Errors | 41 | 43 | 30 | 31 | 0.0487 | 0.39 | 0.2218 |
| **6** | Retrospective Studies # | 465 | 588 | 605 | 774 | -0.0363 | 0.0536 | 0.0092 |
| **7** | Result Presentation | 263 | 320 | 325 | 490 | 0.0957 | 0.2177 | 0.1586 |
| **8** | Result Interpretation | 104 | 320 | 84 | 490 | 0.091 | 0.2168 | 0.1536 |
| **9** | Basic Science Studies# | 52 | 588 | 62 | 774 | -0.0221 | 0.0403 | 0.0083 |
| **10** | Non-Randomised Studies # | 27 | 588 | 54 | 774 | -0.0029 | 0.0495 | 0.0238 |
| **11** | Registration of clinical trials | 12 | 43 | 24 | 41 | 0.0777 | 0.497 | 0.3063 |
| **12** | RCT Sample Size Estimation | 39 | 43 | 31 | 41 | -0.0283 | 0.3251 | 0.1509 |
| **13** | No statistics when needed | 79 | 588 | 111 | 774 | -0.0298 | 0.0466 | 0.0091 |
| **14** | T-Test | 28 | 58 | 39 | 159 | 0.0858 | 0.3854 | 0.2375 |
| **15** | Contingency Tables | 24 | 83 | 52 | 186 | -0.1065 | 0.1371 | 0.0096 |
| **16** | Rank Transformation Methods | 11 | 24 | 12 | 86 | 0.1002 | 0.5372 | 0.3188 |
| **17** | ANOVA | 9 | 16 | 16 | 64 | 0.0261 | 0.5618 | 0.3125 |
| **18** | ANCOVA | 6 | 15 | 2 | 25 | 0.0219 | 0.599 | 0.32 |
| **19** | Regression Analysis | 9 | 13 | 15 | 45 | 0.0172 | 0.6004 | 0.359 |
| **20** | Survival Analysis | 0 | 1 | 10 | 27 | -0.5901 | 0.5753 | 0.3704 |
| **21** | Correlation | 11 | 26 | 15 | 50 | -0.1124 | 0.3592 | 0.1231 |
| **22** | ROC | 1 | 2 | 3 | 17 | -0.2192 | 0.8143 | 0.3235 |
| **23** | Reliability | 1 | 1 | 0 | 12 | 0.0078 | 1 | 1 |
| **24** | Multivariate | 0 | 1 | 2 | 5 | -0.6005 | 0.8296 | 0.4 |
| **25** | Logistic Regression | 3 | 3 | 9 | 43 | 0.0834 | 0.8942 | 0.7907 |
| **26** | Arbitaray p-thresholds | 119 | 320 | 165 | 490 | -0.0335 | 0.1046 | 0.0351 |
| **27** | Reporting p-value with no statistic | 181 | 320 | 397 | 490 | 0.1779 | 0.31 | 0.2446 |
| **28** | T-Test when nonparametric is better | 9 | 58 | 20 | 159 | -0.0712 | 0.1615 | 0.0294 |
| **29** | Association not defined | 70 | 83 | 33 | 186 | 0.5471 | 0.7502 | 0.666 |
| **30** | No Identification of variables used in associated analyses | 56 | 83 | 40 | 186 | 0.3283 | 0.5708 | 0.4596 |
| **32** | Test of association not shown | 39 | 83 | 33 | 186 | 0.1657 | 0.4155 | 0.32925 |
| **33** | One or two tailed? | 78 | 83 | 125 | 186 | 0.1634 | 0.3501 | 0.2677 |
| **34** | No p-value for association | 48 | 83 | 26 | 186 | 0.3102 | 0.5536 | 0.4385 |

**Here,**

kA = Number of articles with said errors in 2003

nA = Total relevant sample size in 2003

kB = Number of articles with said errors in 2013

nB = Total relevant sample size in 2013
